# A Web of Coagulotoxicity: Failure of Antivenom to Neutralize the Destructive (Non-Clotting) Fibrinogenolytic Activity of *Loxosceles* and *Sicarius* Spider Venoms

**DOI:** 10.3390/toxins12020091

**Published:** 2020-01-30

**Authors:** Dwin Grashof, Christina N. Zdenek, James S. Dobson, Nicholas J. Youngman, Francisco Coimbra, Melisa Benard-Valle, Alejandro Alagon, Bryan G. Fry

**Affiliations:** 1 Venom Evolution Lab, School of Biological Sciences, The University of Queensland, St. Lucia, QLD 4072, Australia; d.g.b.grashof@gmail.com (D.G.); christinazdenek@gmail.com (C.N.Z.); j.dobson@uq.edu.au (J.S.D.); n.youngman@uq.edu.au (N.J.Y.); francisco.cp.coimbra@gmail.com (F.C.); 2 Departamento de Medicina Molecular y Bioprocesos, Instituto de Biotecnología, Universidad Nacional Autónoma de México, Av. Universidad 2001, Cuernavaca, Morelos 62210, Mexico; melitza61@gmail.com (M.B.-V.); alagon@ibt.unam.mx (A.A.)

**Keywords:** venom, antivenom, fibrinogen, coagulopathy, spider

## Abstract

Envenomations are complex medical emergencies that can have a range of symptoms and sequelae. The only specific, scientifically-validated treatment for envenomation is antivenom administration, which is designed to alleviate venom effects. A paucity of efficacy testing exists for numerous antivenoms worldwide, and understanding venom effects and venom potency can help identify antivenom improvement options. Some spider venoms can produce debilitating injuries or even death, yet have been largely neglected in venom and antivenom studies because of the low venom yields. Coagulation disturbances have been particularly under studied due to difficulties in working with blood and the coagulation cascade. These circumstances have resulted in suboptimal spider bite treatment for medically significant spider genera such as *Loxosceles* and *Sicarius*. This study identifies and quantifies the anticoagulant effects produced by venoms of three *Loxoscles* species (*L. reclusa*, *L. boneti,* and *L. laeta*) and that of *Sicarius terrosus*. We showed that the venoms of all studied species are able to cleave the fibrinogen Aα-chain with varying degrees of potency, with *L. reclusa* and *S. terrosus* venom cleaving the Aα-chain most rapidly. Thromboelastography analysis revealed that only *L. reclusa* venom is able to reduce clot strength, thereby presumably causing anticoagulant effects in the patient. Using the same thromboelastography assays, antivenom efficacy tests revealed that the commonly used *Loxoscles-*specific SMase D recombinant based antivenom failed to neutralize the anticoagulant effects produced by *Loxosceles* venom. This study demonstrates the fibrinogenolytic activity of *Loxosceles* and *Sicarius* venom and the neutralization failure of *Loxosceles* antivenom, thus providing impetus for antivenom improvement.

## 1. Introduction

Envenomations are complex medical emergencies that can have a range of deleterious effects upon any physiological target reachable by the bloodstream. Symptoms vary widely, depending on many factors, including the offending species. Most venom research has focused on snakes, with spiders being largely neglected due to small venom yields available for testing, as well as fewer reported fatalities from envenomations [1]. In addition, current venom research largely focuses on neurotoxic effects (as reviewed by [2]), as coagulation assays involve inherent difficulties and require working with unstable and expensive coagulation cascade enzymes. As a result of these circumstances, research into the coagulation disturbances produced by spider venoms has been particularly neglected. A lack of knowledge in this regard could lead to uninformed clinical management strategies for spider bites, as well as the persistent use of ineffective antivenoms.

One spider genus known to possess anticoagulant toxins is *Loxosceles*, which includes medically important species such as *Loxosceles laeta* and *L. reclusa* [3]. *Loxosceles* envenomation, or loxoscelism, is the most medically significant spider envenomation in several countries across the Americas, with lethalities on record, and, in particular, constitutes the third highest cause of all accidents by venomous animals in Brazil [4]. Loxoscelism typically results in localized erythema (redness of skin), and/or large areas of ulceration and necrosis [5]. When left untreated this can lead to intensive surgical removal of the dead skin (sometimes requiring skin grafts) and leave behind large scars, thus having economic and emotional impacts [6]. Local loxoscelism effects are a result of the sphingomyelinase D toxin (SMase D), which is a type of phospholipase D toxin in the venom. These dermonecrotic toxins are also the most studied and well-characterized components in *Loxosceles* [7,8].

In addition to local symptoms, in nearly half of the cases, loxoscelism can cause severe systematic medical problems, including hematological disturbances and renal injury, which can progress to hemolysis, thrombocytopenia, shock, disseminated intravascular coagulation, acute renal failure, and even death [1,4,5,6]. These hematological disturbances are most likely induced by metalloproteases, a few of which have been characterized such as Loxolysin A (20–28 kDa) and Loxolysin B (32–35 kDa) found in *L. intermedia* venom [9]. Recent *L. laeta* venom gland transcriptomics identified multiple *Loxosceles* astacin-like metalloproteases (LALPs) within the 20 to 25 kDa range [10]. That study also showed intra-species variation in two *L. laeta* localities (Brazil and Peru): the Peruvian *L. laeta* had an additional LALP at approximately 24 kDa and more glycosylation of LALPs. The hypothesis that the Peruvian *L. laeta* LALPs are more enzymatic compared to the Brazilian *L. laeta* LALPs was confirmed by their fibrinogenolytic activity [10]. These hematological effects are typically noticeable long after the bite incident. These LALPs, similar to the previously described metalloproteases, Loxolysin A and B, were first hypothesized to have digestive functions [11]. However, they may aid in the spread of cytotoxins in the venom by anticoagulant action which exacerbates tissue damage, as has been suggested to convergently occur in some elapid snakes such as spitting cobras (*Naja* spp.) [12,13,14].

Most *Loxosceles* venom studies and bite reports have focused on the necrotic effects of the venom, giving little attention to the systematic anticoagulant effects. Only a few studies have investigated the anticoagulant, fibrinogenolytic (fibrinogen degradation) effects of the venom [3,9,15,16]. Results from 1D SDS-PAGE fibrinogen-cleaving gels have revealed that the venom of multiple *Loxosceles* species cleaves either the Aα chains or the Aα- and Bβ-chains, depending on the study [9] or species [16] with the γ-chain unaffected. Proteomics revealed that multiple LALP isoforms are present, ranging from 24 to 29 kDa, in venoms from three different *Loxosceles* species [17]. By cleaving fibrinogen, fibrinogen levels are lowered and less available for thrombin to cleave into fibrin clots, thereby interfering with blood clotting and producing an anticoagulant effect. This produces coagulopathy, which is observed clinically in loxoscelism cases [5]. However, no studies to date describe the fibrinogenolytic properties of these LALPs across multiple species, nor quantified the extent and speed of fibrinogen cleavage. Similarly, it is unknown if the cleavage of fibrinogen by *Loxosceles* venoms produces a transient, weak clot due to a pseudo-procoagulant mechanism as seen in some snake venoms [18,19,20], or whether destructive cleavage occurs, as seen in some snake venoms [20,21] and anguimorph lizards [22]. Species in the related *Sicarius* genus are known to generate strong necrotic symptoms associated with SMase D-like toxins such as *Loxosceles* [23,24], but their action upon blood coagulation is known.

Therefore, a knowledge gap exists about the potency of *Loxosceles* and *Sicarius* spider venoms upon human blood and the mechanism of coagulotoxic effects, particularly upon fibrinogen. Similarly, a major knowledge gap exists regarding the ability of available antivenoms to neutralize fibrinogenolytic effects of *Loxosceles* venoms. Most studies on *Loxosceles* antivenom, either using crude venom or recombinant toxins, focus on the necrotizing effects of the venom rather than coagulotoxic effects [25,26,27,28,29]. This is reflected in the antivenom production, with the immunizing mixture typically being recombinant spider sphingomyelinase D toxin, not crude (whole) venom due to the impracticality of collecting large quantities of spider venom. For example, Brazil consumes 22,000 ampoules of anti-loxoscelic serum per year, which would require 1.8 kg of venom extracted from approximately 36,000 spiders [8]. As such, to fill venom demand for sufficient antivenom production, antivenom manufacturers are forced to use recombinant toxins in the immunising mixture. While the resulting serum was shown to neutralise sphingomyelinase D [30], the recombinant toxins used in antivenom production represents only a subset of the toxin diversity present within the venoms. Therefore, it is likely that antivenom raised against sphingomyelinase D will not target LALPs nor prevent the coagulation disturbances caused by these toxins. One study showed a polyvalent serum from rabbits using a combination of SMase D toxins, hyaluronidases, and LALPs. The authors suggested that this serum was able to prevent fibrinogen alpha-chain digestion using 100 µL of serum to target 3 µL of *L. intermedia* venom [29]. This high serum:antivenom ratio indicates poor neutralization capacity of this antivenom.

In order to fill the knowledge gaps regarding the fibrinogenolytic activity responsible for coagulopathy, and the relative neutralization by antivenom, this study investigated the fibrinogenolytic effects on human plasma caused by crude venoms from the *Loxosceles* species *L. boneti, L. laeta*, and two different localities of *L. reclusa* (Oklahoma, USA (OK) and Tamaulipas, Mexico (TMP)), as well as the related species *Sicarius terrosus*. Our results have important implications for the clinical treatment of and antivenom treatment for loxoscelism.

## 2. Results

### 2.1. Fibrinogen Digestion

**Figure 1:**
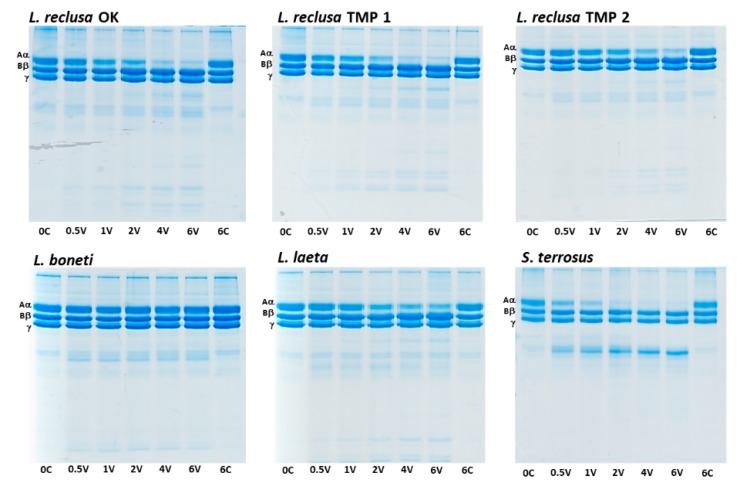
Representative one-dimensional (1D) SDS-PAGE gels showing time-dependent fibrinogen chain degradation (Aα, Bβ or γ) by venom (0.1 μg/μL) at 37 °C over 6 h. C: Negative controls using OK buffer as a blank in place of venom at 0 min and 6 h incubation times; V: Venom incubation over X hours; OK: Oklahoma; USA; TMP: Tamaulipas, Mexico.

**Figure 2:**
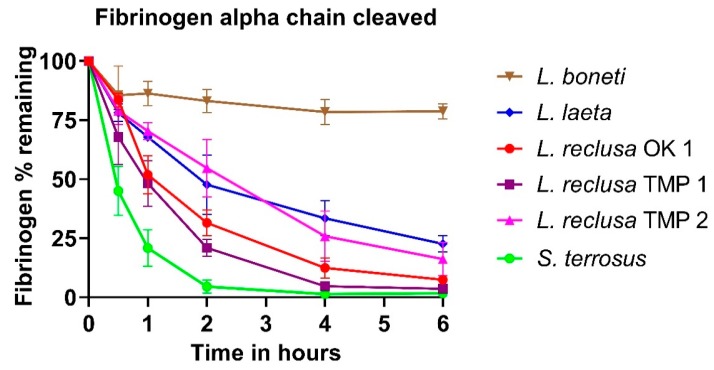
Graphical representation of percentage Aα-chain fibrinogen cleaved using the 1D SDS-PAGE gels from Figure 1. The percentage is derived from the color intensity of the bands, as quantified using ImageJ. Data points are *n* = 3 and the error bars show the standard deviation. OK: Oklahoma; USA; and TMP: Tamaulipas, Mexico.

Fibrinogen gels revealed that some of the venoms efficiently cleaved the Aα-chain of fibrinogen but none of the study venoms cleaved the Bβ- or γ-chains within the time period studied (6 h) (Figure 1). Interspecies variation in venom potency was observed: *S. terrorus* cleaved the Aα-chain most rapidly, whereas all localities of *L. reclusa* and *L. laeta* took longer to cleave the Aα-chain, and *L. boneti* was the slowest and only able to cleave 20% of the Aα-chain (Figure 2). There was also a slight intraspecies variation among the *L. reclusa* localities. The 6 h control allowed us to attribute the Aα-chain digestion to the venom and not natural decay of fibrinogen since no change in fibrinogen was observed in the absence of venom between the 0 min and 6 h. Interestingly, *L. reclusa* OK 1 and *L. reclusa* TMP 1 venoms exerted most of their activity within the first hour (e.g., 50% of the Aα-chain cleaved within an hour) compared to *L. reclusa* TMP 2 and *L. laeta* whose curves have a more gradual cleavage pattern. *Sicarius terrosus* venom cleaved all Aα-chains within a 2 h incubation time with fibrinogen.

### 2.2. Fibrinogen Thromboelastography

**Figure 3:**
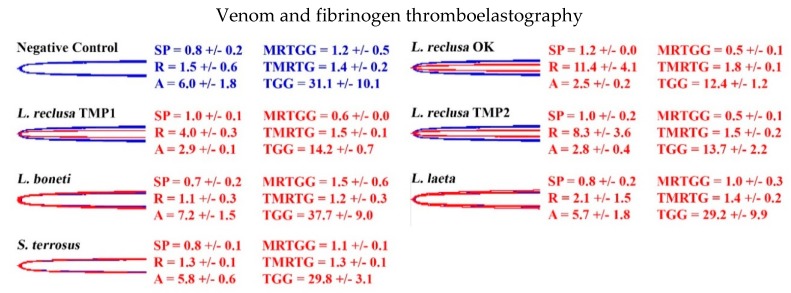
Overlaid thromboelastography traces showing the strength of fibrinogen clots with (red traces) and without (blue traces) venom. Blue traces: Negative controls (thrombin added after 30 min). The wider the trace, the stronger the clot, and vice versa. SP: Split point, time taken until clot begins to form (min); R: Time for initial clot formation where formation is ≥ 2 mm (min); A: Amplitude of clot (mm); MRTGG: Maximum rate of thrombus generation (dynes/cm^2^/s); TMRTG: Time to maximum rate of thrombus generation (min); TGG: Total thrombus generation (dynes/cm^2^). Overlaid traces are *n* = 3 for each set of controls or samples. Values are *n* = 3 means and standard deviation.

Thromboelastography on human fibrinogen revealed that all *L. reclusa* localities slightly weakened the clot strength compared to the negative control (Figure 3). As expected, the venom of *L. boneti* did not differ from the negative control. Interestingly, despite *S. terrosus* venom rapidly cleaving the Aα-chain, thrombin was still able to form a strong clot once added to the cup after the incubation step. Similarly, *L. laeta* venom did not reduce clot strength.

### 2.3. Antivenom Efficacy

**Figure 4:**
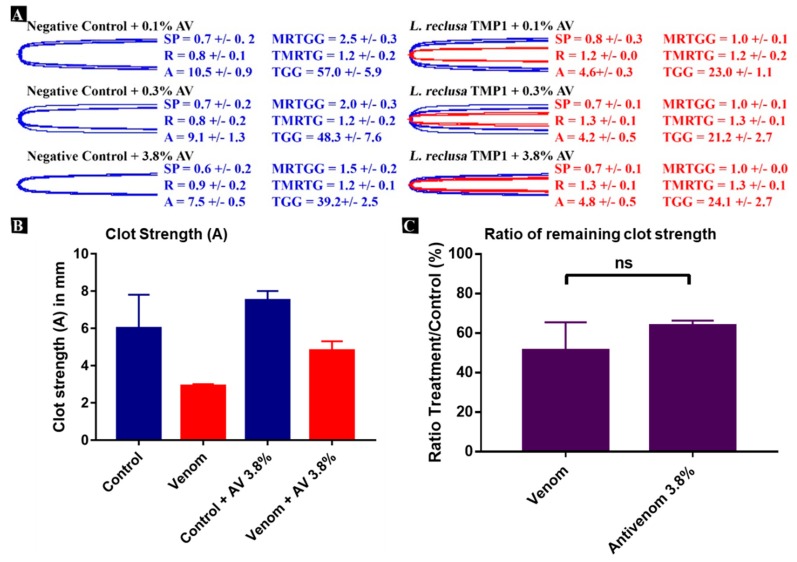
(**A**) Thromboelastography traces of fibrinogen clots produced by spider venom (red) overlaid with spider venom + antivenom (blue), relative to the negative controls (antivenom only). Three concentrations of antivenom were used. SP: Split point, time taken until clot begins to form (mins); R: Time to initial clot formation where formation is > 2 mm (mins); A: Amplitude of clot (mm); MRTGG: Maximum rate of thrombus generation (dynes/cm^2^/s); TMRTG: Time to maximum rate of thrombus generation (min); TGG: Total thrombus generation (dynes/cm^2^). Venom/control overlaid traces are *n* = 3 for each set of controls or samples. Values are *n* = 3 means and standard deviation. (**B**) Overview of clot strength reduction of only venom and venom + AV. Blue bars: Negative control clot strength values from Figure 3 and clot strength values using only 3.8% AV control. Red bars: Clot strength values from *L. reclusa* TMP1 from Figure 3 and *L. reclusa* TMP1 + 3.8% AV. (**C**) Percentage of remaining clot strength relative to control after adding either *L. reclusa* TMP1 venom or *L. reclusa* TMP1 venom + 3.8% AV. The bars show the ratio of intact fibrinogen between the control and venom treatment for the first bar and ratio of intact fibrinogen between the 3.8% AV control and 3.8% AV + venom treatment. TMP: Tamaulipas, Mexico; Ns: Non-significant.

Antivenom was added to the fibrinogenolysis assay on TEG (thromboelastograph) for using three different antivenom end concentrations (0.1%, 0.3%, and 3.8%). Two out of the three antivenom controls had comparable clot strength values of a mean between 10.5 and 9.1 (Figure 4), while the highest concentration has a lower mean of 7.5 +/− 0.5. Using both venom and antivenom all three end concentrations show similar clot strength values of a mean between 4.2 and 4.8 mm (Figure 4A). This is an increase compared to tests without antivenom where *L. reclusa* venoms caused a decrease in clot strength averaged between 2.5 and 2.9 mm (Figure 3).

However, it is also evident that the AV controls on average have stronger clots (compared to samples without the antivenom addition), which decreases in a stepwise manner over increasing AV concentrations. Comparing mean clot strengths of venom and venom + 3.8% AV suggest a minor venom neutralizing effect of the antivenom, but only under the highest antivenom concentration (Figure 4B). These results are indicative of some contribution to fibrinogenolysis by the sphingomyelinase enzymes against which the antivenom is made. On the other hand, the ratio of remaining clot strength shows the slightest observable difference between fibrinogen and venom with or without added antivenom, but statistical tests show no significance between the two with a *p*-value of 0.26 (Figure 4C). This result indicates that a high venom:antivenom ratio of 2:1 is not able to effectively neutralize the venom and reduce its fibrinogenolytic capabilities, consistent with metalloprotease enzymes not being included in the antivenom immunizing mixture.

### 2.4. 1D Gels of Crude Venom

**Figure 5:**
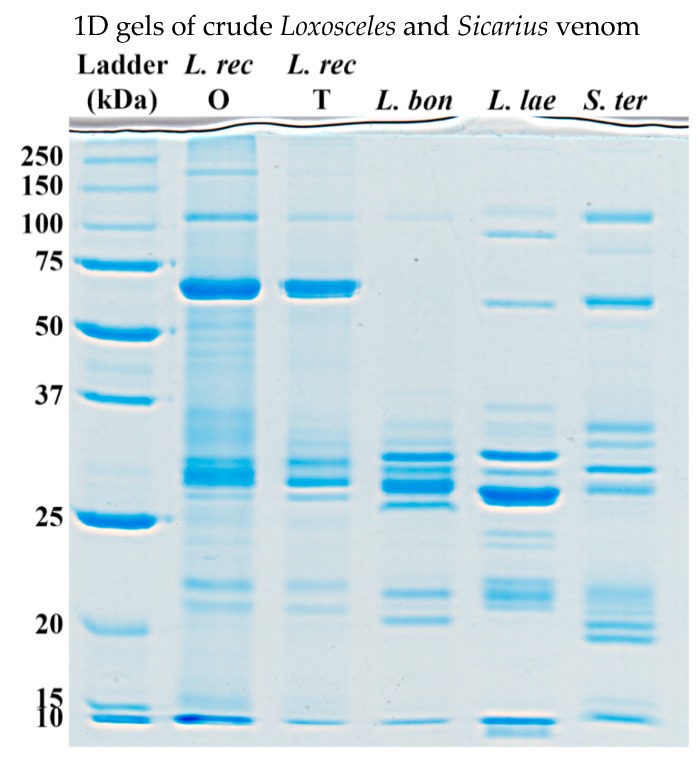
1D SDS-PAGE (reduced) gel comparisons: L: Ladder; rec O: *L. reclusa* OK; L. rec T: *L. reclusa* TMP2; L. bon: *L. boneti*; L. lae: *L. laeta*; S. ter: *S. terrosus*; OK: Oklahoma; USA; and TMP: Tamaulipas, Mexico.

Crude venoms were analyzed by 1D SDS-PAGE gels under reducing conditions (Figure 5). All venoms showed similar banding patterns, except a band in the upper 50–75 kDa range being present in all spiders excluding *L. boneti*, which is the only investigated spider showing no fibrinogenolytic activity in the fibrinogen gels.

## 3. Discussion

Functional activity tests in the form of fibrinogen digestion in gels show the extent of the fibrinogenolytic properties of the venoms. These assays showed that all studied Sicariidae species were able to cleave only the Aα-chain, although *L. boneti* to a low extent. No cleavage activity was observed on the Bβ- or γ-chains for all species. This is congruent with literature where *L. intermedia* was found to cleave the Aα-chain with no effect on the Bβ-chain [9]. However, another study showed *L. intermedia* venom cleaved both the Aα- and the Bβ-chain, which is probably attributed to the relatively long (16 h) incubation period [16] compared to the present studdy (6 h). The extreme fibrinogenolytic effects of *S. terrosus* were unexpected since no previous studies have looked at fibrinogenolytic properties in their venom; however, a review on *Sicarius* sp. bites does state cases of widespread haemmorrhage [31], which may indicate fibrinogenolytic activity. The limited ability of *L. boneti* venom to cleave fibrinogen seems congruent with the absence of bite reports stating systematic bleeding, as this absence of bite reports suggest that *L. boneti* venom does not cause systemic bleeding that requires clinical treatment. This is in contrast with *L. reclusa*, since the observed fibrinogenolytic effects from *L. reclusa* venom are congruent with observed patient symptoms such as hemolysis, disseminated intravascular coagulopathy, and systematic bleeding as seen in confirmed *L. reclusa* bite reports [32].

The differential fibrinogenolytic functional effects of the venoms was reflected in fibrinogen gel degradation products. Where the *Loxosceles* venom produces multiple faint degradation product bands on 1D gels, *Sicarius terrosus* produces only one intense band (Figure 1). This indicates a difference in cleaving sites and activity of the responsible toxin and thus probably also the nature and severity of the symptoms. This could be very important for *S. terrosus* bite treatment with *Loxosceles*-specific antivenom since there is no specific *S. terrosus* antivenom in production. However, a recent study does show cross-reactivity of heterophilic antibodies for loxoscelism between multiple *Loxosceles* species and *Sicarius terrosus* [33].

Thromboelastography assays revealed that while *L. reclusa* had a moderate fibrinogenolytic activity of the Aα-chain in the fibrinogen gels, it produced a greatly reduced clot strength in TEG assays. In contrast, while *S. terrosus* showed relatively potent and fast fibrinogenolytic activity on the Aα-chain in the fibrinogen gels, it failed to reduce the clot strength in TEG assays. Inconsistency between fibrinogen gels and thromboelastography has been shown before within the varanids: the authors hypothesized that the speed of cleaving Aα-chains and presumably Bβ-chains explained the reduced clot strength [22]. However, *S. terrosus* venom cleaves the Aα-chain at a higher speed than *L. reclusa* venom and this is therefore unlikely to be the underlying reason for this observed inconsistency. Another explanation could be the cleaving site of their toxins and therefore, more importantly, the degradation product(s) of the reaction. As *S. terrosus* venom gives a single observable degradation product, compared to *L. reclusa* venom producing more complex (and thus faint) degradation products in the fibrinogen gels, this suggests that the fibrinogenolytic toxins in *S. terrosus* venom may cleave the Aα-chain in such a manner that it does not inhibit thrombin from producing a strong clot. 

It is important to note that the Aα-chain cleaving venom alone will not produce coagulation symptoms, as cleaving just the Aα-chain alone does not produce a fibrin clot [34,35,36]. Whereas, *L. reclusa* venom most likely cleaves the Aα-chain in a destructive manner at multiple cleavage sites, thus preventing the resulting fibrin strands from being able to form a clot. The local hemorrhagic lesions caused by *S. terrosus* venom should, therefore, be explained, for example, by blood vessel damage caused by phospholipase D toxins also present in the venom.

To test for the ability of the SMase D-specific antivenom to neutralize the fibrinogenolytic toxins in the studied Sicariidae venoms, antivenom was added to thromboelastography assays. These assays revealed a minor increase of clot strength compared to *L. recluse* venom but only across all three concentrations of antivenom, indicating some cross-reactivity within the venom. The increasing ratio of venom:antivenom did not further increase the clot strength, even at a 2:1 venom:antivenom ratio. This is nevertheless unlikely to be a clinical increase in clot strength as shown by the ratio of remaining clot strength between the controls and the venom/venom:antivenom. Statistical tests show that this increased clot strength ratio of the venom:antivenom is not significantly different from the venom clot strength ratio, even more so indicating that this antivenom has no clinical neutralizing effect on the fibrinogenolytic toxins. This is not surprising since the antivenom was made using recombinant SMase D toxins, which are thought to not show any fibrinogenolytic activity. This assay revealed that, despite significant reductions described in loxoscelism using recombinant SMase D antivenom [28,30,37], those antivenoms are unlikely to provide any neutralization of fibrinogenolytic toxins and their associated systematic effects.

Surprisingly, the highest concentration of antivenom in the negative control showed a decrease in clot strength, where clot strength was negatively correlating with the volume of antivenom added to the sample on TEG. While the average strength of clots were overall higher with antivenom than the controls were without antivenom, the average clot strength did decrease in the controls as antivenom concentration increased. To our knowledge, this is the first time clot strength was measured to test any adverse effects that the antivenom itself might have on the clot strength of fibrinogen. However, since the concentration of antivenom in our tests was so high, it is unlikely that similar adverse effects in a clinical setting could be produced, as such antivenom levels would be exceedingly unlikely to be reached [38]. Thus, while biochemically interesting, these effects are not likely to be clinically relevant. The effects are likely due to a preservative or other stabilizing chemicals that have a nonspecific effect upon the fibrinogen due to the very high concentrations used in the last tests.

1D gels of Sicariidae venoms were congruent with those of previously studied crude venoms [23]. Gel bands across different species revealed major interspecific variation, and some minor intraspecific variation between the two different localities (*L. reclusa* OK and *L. reclusa* TMP) were also evident. Previous venom characterizations of related Sicariidae spiders focused on toxins in the 5–40 kDa range, which were reported to be a complex mixture of phospholipases-D, astacin-like metalloproteases, and inhibitor cystine knot (ICK) peptides [8,17]. This is congruent with 1D gels presented in this study, showing most intense bands in the 25–37 kDa range and fainter bands in the 20–25 kDa range for all species. Even more so, literature characterized most toxins in the 25–37 kDa range as phospholipase-D or metalloproteinase subtype Loxolysin B, and toxins in the 20–25 kDa range as metalloproteinase subtype Loxolysin A. This distinction in range categories is also visible in the gels obtained in this study. Of which Loxolysin B is previously shown using SDS-PAGE gels to have fibrinogenolytic activity [9]. Higher molecular weight bands in the 50–75 kDa range which largely correlate with the venom’s ability to cleave fibrinogen Aα-chains (e.g., only present in *L. reclusa*, *S. terrosus,* and faintly in *L. laeta*) are characterized as either zinc metalloproteinases, found in the venom gland transcriptome of *L. intermedia*, or high molecular weight serine-proteinases, isolated from *L. intermedia* venom [39]. However, isolated serine-proteinases from *L. intermedia* venom were shown to have no fibrinogenolytic activity, making it likely that fibrinogenolysis is due to zinc metalloproteinases. Without protein identification and fractionation no definitive conclusions can be made about the underlying toxin or toxins causing the observed fibrinogenolytic effects of some of these Sicariidae venoms. Therefore, future studies should test fibrinogenolytic activity of Sicariidae venom fractions, as well as sequence the fibrinogen degradation products to elucidate any variability in fibrinogen splice sites.

Despite *Loxosceles* spiders bites resulting in complex medical emergencies [32,40] the effects of spider venoms upon coagulation has been a neglected area of research. This study demonstrates the severity of and significant interspecies variation in fibrinogenolytic activity for four Sicariidae spider species, with *L. reclusa* also showing destructive (non-clotting) fibrinogenolytic effects by reducing clot strength. The available antivenom was unable to neutralize fibrinogenolytic toxins present in these spiders, thereby drawing attention to this functional activity and the treatment plans for the resulting systematic effects. In conclusion, this study provides data relevant to clinical treatment of envenomed patients, particularly regarding the source of systematic afibrinogenemia effects and the absence of proper systematic-loxoscelism treatment to prevent these possibly deadly effects.

## 4. Materials and Methods

### 4.1. Venom Samples

Spider species represented in this research were *Loxosceles reclusa* (one sample from Oklahoma, USA and two samples from Tamaulipas, Mexico, referred to as OK and TMP 1 and 2, respectively), *L. boneti, L. laeta,* and *Sicarius terrosus*. Freeze-dried venom from *L. reclusa* (gland extracts) and *L. boneti* (milked venoms) were obtained from the UNAM collection of author AA and the venom from *L. laeta* and *S. terrosus* was obtained from VenomTech and stored at −80 °C until use. Venom aliquots were solubilized in cold deionized water and centrifuged for 15 min at 4 °C (14,000 RCF). Afterward, the supernatant was collected and protein concentrations measured using a ThermoFisher Scientific (Waltham, MA, USA) NanoDrop™ spectrophotometer. Working stocks of 1 mg/mL were made with 50% deionized water and 50% glycerol, and then stored at −20 °C.

### 4.2. Human Fibrinogen

Human fibrinogen (Lot#F3879, Sigma Aldrich, St. Louis, MO, USA) was reconstituted in an enzyme running buffer (150 mM NaCl, 50 mM Tri-HCl (pH 7.3)) to a concentration of 4 mg/mL. The solution was then flash-frozen in liquid nitrogen as 1 mL aliquots and stored at −80 °C until required, where it was then defrosted in a water bath at 37 °C for 5 min.

### 4.3. Antivenom

In this study, we used the AAA anti-arachnid antivenom (Study drug: AAA2013-BB-IND 15757; Batch number 3IT08001; protein concentration of 18.83 ± 0.03 (*n* = 3)). As per manufacturer’s instructions, the antivenom was made up in 2 mL deionized water and inverted until dissolved completely. Antivenom was centrifuged (10 min, 4 °C, 14,000 RCF), and the supernatant collected stored at 4 °C. Antivenom working stocks (2.6% and 7.5% antivenom) were made by diluting antivenom with an Owren Koller (OK) Buffer (Stago catalog #00360) and then stored at 4 °C.

### 4.4. Fibrinogen 1D SDS-PAGE Electrophoresis and Gel Image Analysis

The ability of the spider venoms to cleave fibrinogen chains was investigated by using an adapted protocol previously published by this lab [41,42,43]. Details of the gel are as follows: 3.3 mL deionized water, 4.0 mL 30% acrylamide mix, 2.5 mL 1.5 M Tris-HCl, pH 8.8, 100 µL 10% SDS, 100 µL 10% APS, and 4 µL TEMED (resolving/running gel); 1.4 mL deionized water, 330 µL 30% acrylamide mix, 250 µL 0.5 M Tris-HCl, pH 6.8, 20 µL 10% SDS, 20 µL 10% APS, 2 µL TEMED (stacking gel). Resolving gel was cast first and the running gel was added after the resolving gel was solidified.

Spider venom and fibrinogen were prepared as described above. For this assay 43 µL of 4 mg/mL fibrinogen was mixed with 107 µL of an enzyme running buffer, making it 150 µL of 1 mg/mL fibrinogen. Of that, 10 µL was aliquoted for the 0 min control and 20 µL was aliquoted and incubated for 6 h at 37 °C as a 6 h control to account for any evaporative loss. To reach a final ratio of 1:10 venom/fibrinogen in the assay, 13.3 µL (133/10 = 13.3) of 1 mg/mL venom was added. The sample was then incubated at 37 °C for 6 h while 10 µL of the sample was aliquoted out for each of the following time points: 30 min, 1, 2, 4, and 6 h. 10 µL of the aliquoted sample was added to 10 µL of buffer dye (5 µL of 4 × Laemmli sample buffer (BioRad, Hercules, CA, USA) and 5 µL of 100 mM DTT (Sigma-Aldrich, St. Louis, MO, USA) in deionized water). This was also done for the 0 min and 6 h controls which serve as untreated control lanes. Each aliquot was boiled at 100 °C for 4 min, directly after it was mixed with the buffer dye. Furthermore, a venom-only control lane was made by adding 6 µL of 1 mg/mL venom to 54 µL of an enzyme running buffer for a final ratio of 1:10 venom/enzyme running buffer. 10 µL of the venom control was added to 10 µL of buffer dye and boiled the same way as the other aliquots. Each venom was tested in triplicate, resulting in three separate gels per venom used for analysis.

The aliquots were then loaded into a 1D SDS-PAGE gel using the following set up: Lane 1: 0 min untreated fibrinogen control; Lane 2: 30 min venom: + fibrinogen incubation; Lane 3: 1 h venom + fibrinogen incubation; Lane 4: 2 h venom + fibrinogen incubation; Lane 5: 4 h venom + fibrinogen incubation; Lane 6: 6 h venom + fibrinogen incubation; Lane 7: 6 h untreated fibrinogen control incubation;. Gels were run in 1 × gel running buffer at room temperature at 120 V until the dye neared the bottom of the gel cast [18,43,44]. All gels were run in triplicate under the same conditions. Afterwards, gels were stained using colloidal Coomassie brilliant blue G250 (34% methanol (VWR Chemicals, Tingalpa, QLD, Australia), 3% orthophosphoric acid (Merck, Darmstadt, Germany), 170 g/L ammonium sulfate (Bio-Rad, Hercules, CA, USA), 1 g/L Coomassie blue G250 (Bio-Rad, Hercules, CA, USA), and destained overnight using deionized water.

Gels were analyzed using the publicly available software ImageJ (V1.51r, Java 1.6.0_24, National Institutes of Health, Bethesda, Maryland, USA) [45]. To quantify fibrinogen cleavage, all gel images were scanned using a standard printer/scanner and loaded into ImageJ. Images were changed to 32-bit, and bands were emphasized using the ‘Adjust Brightness/Contrast’ option. To quantify the intensity of the bands, a box was drawn over the first control and was selected as the first lane using ‘Analyze -> Gels -> Select first lane’. Sequential lanes were selected by placing boxes on the remaining lanes and using the ‘Analyze -> Gels -> Select next lane’ option, and the intensity of each band was plotted using the ‘Analyze -> Gels -> Plot lanes’ function. The individual peaks (representing the intensity of the Aα-, Bβ-, and γ- chains, respectively) were separated with the “Draw line” function and quantified using the “Wand” function on each peak which produces an area under the curve value representing the intensity of a band. These values were entered into Windows Excel 2016, the average taken of triplicates, and the data graphed with standard deviation using GraphPad PRISM 7.0 (GraphPad Prism Inc., La Jolla, CA, USA).

### 4.5. Thromboelastography

To test the ability of venoms to reduce clot strength, a Thrombelastograph^®^ Haemostasis System 5000 (Haemonetics^®^, Haemonetics Australia Pty Ltd., North Ryde, Sydney 2113, Australia) was used in combination with Version 4 TEG Analytical Software (TAS^TM^). This machine measures the elasticity, strength, and stability of the formed clot using a cup and pin, where the forming clot in the rotating cup creates tension on a torsion wire inside a fixed pin which can be transduced into values used to measure the clot. Important values are SP: Split point, time taken until clot begins to form (min); R: Time to initial clot formation where formation is ≥ 2 mm (mins); A: Amplitude of clot (mm); MRTGG: Maximum rate of thrombus generation (dynes/cm^2^/s); TMRTG: Time to maximum rate of thrombus generation (min); TGG: Total thrombus generation (dynes/cm^2^). Assays were adapted from previous published protocols [18,19,21,42].

To maintain similar ratios as previously validated protocols, for this assay 200 µL of 4 mg/mL fibrinogen was added to 86.4 μL CaCl_2_ (25 mM stock solution Stago catalog #00367 STA), 86.4 μL phospholipid (solubilized in OK buffer adapted from STA C·K Prest standard kit, Stago catalog #00597), 16 μL OK buffer, and 32.4 µL of 1 mg/mL venom 50% glycerol stock or 32.4 µL 50% deionized water/glycerol as a negative control. While the ratio of venom:fibrinogen was not identical to the ratio used in the fibrinogen gels (1:10), 1:6 (200/32.4 = 6.17) was the maximum ratio for this experimental setup due to cofactor and antivenom volume constraints in the assay. The total volume was 421.2 µL, with fibrinogen at a final concentration of 1.9 mg/mL and venom at a concentration of 0.3 mg/mL. These mixtures were then incubated at 37 °C for 2 h, which was based on results from previous fibrinogen 1D SDS-PAGE gels. After incubation, 360 µL was transferred to ‘natural pins and cups’ (Lot# HMO3163, Haemonetics Australia Pty Ltd., North Ryde, Sydney 2113, Australia) and 7 µL of thrombin was added (from Stago Liquid Fib kit, unknown concentration from supplier (Stago catalog #115081 Liquid Fib)) to force clotting of the remaining fibrinogen which was measured over a time period of 30 min.

### 4.6. Antivenom Efficacy and Statistics

In a similar fashion as the clotting inhibition tests, these assays used the Thrombelastograph^®^ 5000 Haemostasis analyser (Haemonetics^®^, Haemonetics.com, Cat# 07-033) as well. This assay used 200 µL of 4 mg/mL fibrinogen, 86.4 μL CaCl_2_, 86.4 μL phospholipid, 32.4 µL of 1 mg/mL venom 50% glycerol stock or 32.4 µL 50% deionized water/glycerol as a negative control, and 16 µL of previously mentioned 2.6%, 7.5%, or 100% antivenom, resulting in 0.1%, 0.3%, and 3.8% antivenom concentration in the end, respectively. The solution was incubated at 37 °C for 2 h. Afterward, 360 µL was transferred to the cup and 7 µL of thrombin was added. Clot-formation parameters were automatically measured over a period of 30 min.

Significance between the venom-only treatment and venom + antivenom treatment was calculated in GraphPad PRISM 7.0 using a t-test. A Shapiro–Wilk normality test gave a *p*-value of 0.66 for the venom treatment and 0.93 for the venom and antivenom treatment. These *p*-values show that the values were not significantly different than a Gaussian distribution, allowing the use of a t-test. Assuming Gaussian distribution, we performed an unpaired t–test with Welch’s correction for unequal standard deviations.

### 4.7. 1D Gels of Crude Venom

In order to investigate the proteomic variation in venoms, 1D gradient gels were run under both reducing and nonreducing conditions using the same gel setup as described above in Section 4.4.

25 µg of venom was diluted in 7.5 µL of deionized water and 7.5 µL of 2 × Laemmli sample buffer for nonreduced venom and in 7.5 µL of deionized water and 7.5 µL of 50% 2 × Laemmli sample buffer and DTT for reduced samples. Reduced samples were boiled for 4 min at 100 °C. Nonreduced and reduced samples were loaded on different gels and both gels run with 8 µL of unstained ladder. Afterward, gels were stained using colloidal Coomassie brilliant blue and destained overnight using deionized water.
